# Phytochemical Characterization of Chamomile (*Matricaria recutita* L.) Roots and Evaluation of Their Antioxidant and Antibacterial Potential

**DOI:** 10.3390/molecules27238508

**Published:** 2022-12-03

**Authors:** Lilo K. Mailänder, Peter Lorenz, Hannes Bitterling, Florian C. Stintzing, Rolf Daniels, Dietmar R. Kammerer

**Affiliations:** 1 Department of Analytical Development and Research, Section Phytochemical Research, WALA Heilmittel GmbH, Dorfstraße 1, DE-73087 Bad Boll/Eckwälden, Germany; 2 Department of Pharmaceutical Technology, Tübingen University, Auf der Morgenstelle 8, DE-72076 Tübingen, Germany

**Keywords:** *Matricaria chamomilla* L., *Matricaria discoidea* DC., phytoextract, HPLC-MS, GC-MS, bioactive constituents, biological activity

## Abstract

*Matricaria recutita* L., German chamomile, is one of the most widely used medicinal plants, whose efficacy has been proven in numerous studies. However, its roots have attracted only little interest so far, since mainly above-ground plant parts are used for medicinal purposes. To broaden the knowledge of chamomile roots, a profound phytochemical characterization was performed along with a bioactivity screening of corresponding root extracts. While volatile constituents such as chamomillol and polyynes were detected using GC-MS, HPLC-MS^n^ analyses revealed the occurrence of four coumarin glycosides, more than ten phenolic acid esters and five glyceroglycolipids. Furthermore, the antioxidant activity of the extracts was evaluated. Polar extracts revealed IC_50_ values ranging from 13 to 57 µg/mL in the DPPH radical scavenging assay, which is in the same range as reported for chamomile flower extracts. In addition, superoxide radical scavenging potential and mild antibacterial effects against *S. aureus* und *B. subtilis* were demonstrated. Moreover, to assess interspecies variation in chamomile roots, extracts of *M. recutita* were compared to those of *M. discoidea* DC. Interestingly, the latter revealed stronger antioxidant activity. The presented results aim at the valorization of chamomile roots, previously discarded as by-product of chamomile flower production, as a sustainable source of bioactive phytochemicals.

## 1. Introduction

*Matricaria recutita* L., also known as German chamomile, is an annual plant belonging to the *Asteraceae* (*Compositae*) family. It has yellow-white flowers, bi- to tripinnate leaves, and can be distinguished from related species by its hollow flower heads. Originating from Southern and Eastern Europe, chamomile is now widespread from Europe to India, throughout America as well as in Australia and New Zealand [1,2]. Chamomile is among the most important medicinal plants [3] with a production quantity of 7000–8000 tons per year [2]. For this reason, the secondary metabolite profile of aerial parts, especially flowers, together with their antioxidant, antimicrobial and pharmacological activities have been extensively studied in vitro and in vivo and remain a current research topic [4,5]. Infusions of chamomile are among the most consumed single-ingredient herbal teas [6] and, according to the European Medicines Agency monograph, are used for the treatment of gastrointestinal, mouth, throat, and skin disorders, minor wounds, or colds [7]. The beneficial effects are mainly attributed to the presence of phenolic compounds, such as apigenin-7-glucoside or hydroxycinnamic acid derivatives [8]. Moreover, alcoholic chamomile extracts have been proven to show cardioprotective, neuroprotective, antispasmodic and antitumor effects [5]. The dark blue essential flower oil contains chamazulene, which is derived from the sesquiterpene lactone matricin during distillation. Furthermore, sesquiterpenoids such as farnesene, *α*-bisabolol and its oxides and acetylene derivatives such as polyynes have been detected in the essential oil. It has spasmolytic, anti-inflammatory and antiseptic activities and is often applied for cosmetic purposes [1]. Depending on the ratios of *α*-bisabolol and the bisabolol oxides A and B in the essential flower oil, chamomile cultivars are assigned to different chemotypes [9].

Besides *M. recutita*, other *Matricaria* species are occasionally used in folk medicine. For instance, the flowers of *M. discoidea* (pineapple weed) have a strong chamomile odor, but lack the white petals. The aerial parts of this species contain about 10% polyphenols, among others hydroxycinnamic acid derivatives, and the coumarins herniarin and umbelliferone [10]. *β*-Farnesene, geranyl-isovalerate and the (Z)-spiroether are the main components of *M. discoidea* essential oil [11]. Cantrell et al. demonstrated its strong insect-repellent activity [12]. *M. aurea* (golden chamomile) is another species used for medicinal purposes, the extracts of which exhibit antioxidant activity, inhibit the growth of *Bacillus subtilis* and *Staphylococcus aureus,* and even show antiproliferative activities on cancer cells [13]. Last but not least, *M. pubescens* (hairy chamomile), which is used in traditional Algerian medicine, contains similar flavonoids as *M. recutita*. It exhibits a protective effect against mild toxic doses of UV-A light on 3T3 fibroblasts [14].

In the 1st century AD, Dioscorides recommended not only decoctions of chamomile flowers, but also of the herb and roots as tonic and for treating urinary tract disorders, i.e., inflammation, spasms, ulcers. Topical applications included the treatment of wounds and burns. Furthermore, Dioscorides prescribed chamomile suppositories against recurrent fever [15,16]. Nowadays, aqueous fermented extracts prepared from chamomile roots are still used in complementary medicine. Indications are similar to those of flower preparations, i.e., the treatment of cramps, gastrointestinal and biliary problems, flatulence, menstrual cramps, teething problems, and sleep disorders of young children [17].

Due to their limited use, only few studies on chamomile roots have been reported. Early investigations showed that they contain 0.04–0.09% essential oil, which is localized in oil cells in the root cortex [16,18,19]. This pale yellow oil is mostly obtained by steam distillation in a Clevenger-type apparatus. It contains up to 45% *β*-farnesene and various other sesquiterpenes, but is devoid of bisabolol and chamazulene [19,20]. The content of chamomillol in essential root oil increases from early growth stages until the end of flowering, although chamomillaester and spiroether contents decrease [18]. In aqueous chamomile root extracts, cinnamic and benzoic acid derivatives such as chlorogenic, caffeic, ferulic, protocatechuic, vanillic and syringic acids were detected by HPLC-MS in concentrations of 1.5–20.4 µg·g ^−1^ dry weight [21]. Further investigations into chamomile roots have focused on the impact of abiotic stress factors from an agricultural perspective. As an example, nitrogen deficiency enhances root growth and total phenolic accumulation as it suppresses soluble protein contents [22]. Chamomile is a known heavy metal accumulator. Although copper accumulation causes oxidative stress and leads to increased malondialdehyde concentrations in the roots [23,24], chamomile is tolerant to high cadmium concentrations [25]. Further investigations into chamomile roots, especially a comprehensive phytochemical characterization and an evaluation of their bioactivity profile, have not yet been conducted. Therefore, the present study focused on a broad GC-MS and HPLC-DAD-MS^n^ screening of secondary metabolites in mid-polar and polar *M. recutita* and *M. discoidea* root extracts. Furthermore, their antioxidant potential as well as antibacterial activity against the Gram-positive bacteria *B. subtilis* and *S. aureus* were assessed.

## 2. Results and Discussion

### 2.1. Secondary Metabolites in M. recutita Roots at Different Developmental Stages

**Figure 1:**
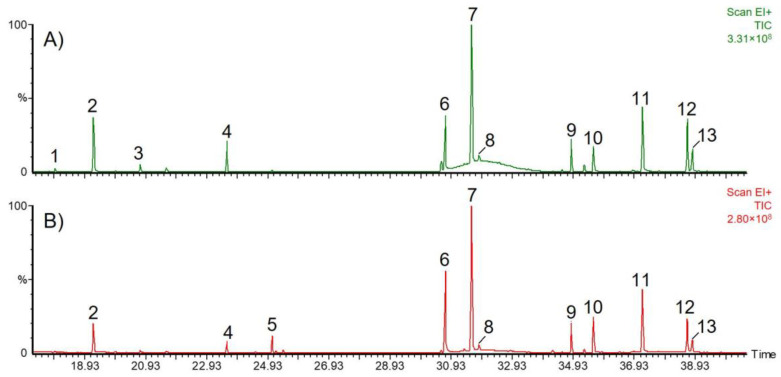
GC-MS total ion current chromatograms of *M. recutita* root dichloromethane extracts after silylation. Roots harvested (**A**) in March and (**B**) in June. Peak numbers refer to Table 1.

**Figure 2:**
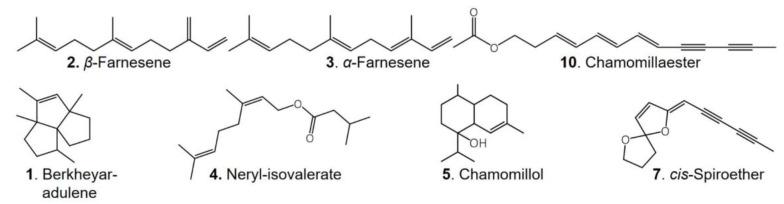
Structures of selected representatives characterized in *M. recutita* root dichloromethane extracts.

For GC-MS analyses, essential root oils obtained by steam distillation were analyzed in *n*-hexane/ethyl acetate. DCM extraction of fresh roots yielded 0.20% (m/m) of a highly viscous residue, which was dissolved in chloroform at concentrations of 5 mg/mL for direct analysis, or derivatized to obtain trimethylsilyl esters. The compound profiles of volatile secondary metabolites were identical in essential root oils and DCM extracts. Most volatile constituents were assigned by GC-MS analysis through their retention times and MS data, which were compared with the NIST database (National Institute of Standards and Technology, match factor > 800). A typical chromatogram together with the assigned compounds is displayed in Figure 1 with the corresponding mass spectral data being displayed in Table 1. Figure 2 illustrates structures of typical representatives of such extracts.

The sesquiterpenes berkheyaradulene (compound **1**), *β*-farnesene (**2**) and *α*-farnesene (**3**) were detected besides neryl-isovalerate (**4**) and traces of other terpenes. Terpenoids are prevalent in the plant kingdom, where they serve as plant hormones and signaling molecules. For example, they are often released upon damage of plant tissues in order to induce defence mechanisms. Terpenoid composition and concentration may vary substantially depending on the growth stage [26]. High amounts of farnesene are presumably due to premature harvesting [4]. Indeed, we found that farnesene concentration in DCM extracts decreased by about half from March to June. Chamomillol (**5**) was identified upon comparison of its fragmentation pattern with that published by Reichling et al. [18], who demonstrated an increase in the content of this sesquiterpene alcohol in chamomile roots from early growth stages until the end of flowering. Accordingly, we detected this compound in roots harvested in May and June, just before and during flowering, but not in March and April. Compound **6** was tentatively assigned to a sesquiterpene oxide. Its fragmentation pattern, however, does not correspond to that of caryophyllene oxide, which has previously been identified in chamomile roots [18]. Further, two spiroether isomers could be distinguished by their retention times. Both compounds were assigned based on the fact that the *cis* isomer (**7**) is more abundant than the *trans* isomer (**8**) [18,27]. In addition, the trimethylsilyl esters of palmitic (**9**), linoleic (**12**) and linolenic acids (**13**) were identified after derivatization of the extract compounds with *N*,*O*-bis (trimethylsilyl)-trifluoroacetamide (BSTFA). Compounds **10** and **11** revealed an M^+^ ion at *m*/*z* 228, which could not be further characterized. Based on their molecular mass and fragmentation patterns, these two substances were assigned to chamomillaester I and II, which have been previously described in *Matricaria* roots [18,28]. Although Das et al. reported the occurrence of bisabolol and its oxides in essential root oil [19], those compounds were detected neither in our investigations nor in those of Reichling et al. [18].

**Figure 3:**
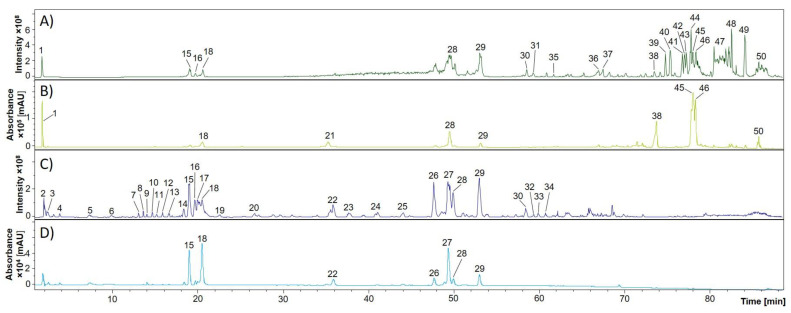
Secondary metabolites in root extracts of *M. recutita* analyzed via RP-HPLC-DAD-ESI-MS^n^. The peak numbering corresponds to Table 2. (**A**) Base peak chromatogram (BPC) of an ethyl acetate extract; (**B**) Corresponding UV chromatogram (200–600 nm); (**C**) BPC of an *n*-butanol extract; (**D**) Corresponding UV chromatogram (200–600 nm). Peak numbers refer to Table 2.

The yields of EtOAc and *n*-BuOH extractions were 0.05% and 0.12% (*m*/*m*), respectively. For HPLC-DAD-MS^n^ analyses, plant extracts were dissolved in purified water or methanol. Individual metabolites were characterized based on their retention times, UV spectra and fragmentation behavior in comparison with literature data or analytical standards. Base peak and UV chromatograms of representative EtOAc and BuOH extracts (March harvest) are illustrated in Figure 3 and peak assignment is displayed in Table 2.

**Figure 4:**
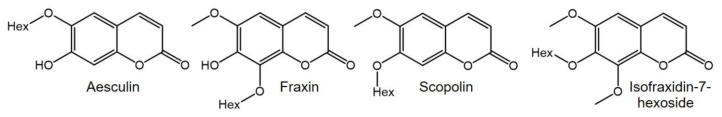
Structures of coumarin hexosides detected in *M. recutita* roots.

A number of compounds with similar fragmentation patterns and UV spectra were eluted in a retention time range of 15–21 min. Based on neutral losses of 162 Da resulting in [M−H−hexosyl]^−^ ion species in the first fragmentation step and the mass-to-charge ratios of the corresponding aglycons, four coumarin glycosides, namely aesculin (compound **12**, t_R_ 15.6 min, *m*/*z* 339), scopolin (**14**, t_R_ 18.4 min, *m*/*z* 399), fraxin (**16**, t_R_ 19.7 min, *m*/*z* 369), and isofraxidin-7-glucoside (**18**, t_R_ 20.6 min, *m*/*z* 383) were assigned (Figure 4). The identity of aesculin and fraxin was verified using analytical reference standards. Compounds **11** and **17** revealed losses of 80 Da (sulfate or phosphate) upon collision-induced dissociation (CID). Since coumarin sulfates have been described earlier in *Pelargonium* species [55] and are formed in coumarin metabolism [56], the two substances were tentatively assigned to fraxin and fraxetin sulfate. Additionally, neutral losses of 208 Da for compounds **13** and **20** indicated the presence of further fraxetin derivatives. However, these could not be more closely identified. The coumarins herniarin, umbelliferone, esculetin, scopoletin and daphnetin, together with some of their glycosides, have previously been detected in chamomile flowers [57,58]. To the best of our knowledge, coumarins in general have not been detected in chamomile roots so far, and also fraxidin and fraxetin in *M. recutita* are described here for the first time. This is of particular interest, since in the plant kingdom coumarins play a role in iron uptake and bioactivities reported in in vitro studies are, among others, antimicrobial and anticoagulant [59].

Furthermore, a number of caffeoylquinic acids (CQA) were characterized in EtOAc and BuOH extracts. These show interesting bioactivity characteristics, such as antiphlogistic and enzyme-inhibiting properties [60]. Molecular ions at *m*/*z* 353 with intense signals at *m*/*z* 191 in MS^2^ experiments (compounds **10, 15, 19**) indicated the presence of 3-, 4- and 5-*O*-chlorogenic acids, respectively. For compounds **21** and **26**–**30**, fragmentation of the [M−H]^−^ ions at *m*/*z* 515 yielded daughter ions at *m*/*z* 353 ([M−H−162]^−^, loss of caffeoyl moiety). Together with UV maxima at 218 and 322 nm, the compounds were assigned to different isomers of dicaffeoylquinic acids (diCQA). The constitutional isomers were differentiated based on their MS^2^ and MS^3^ fragment ion intensities according to Clifford et al. [44]. The occurrence of mono- and diCQA in chamomile roots has been reported previously [60]. A decrease in diCQA contents was found to be the main difference between plants of various growth stages from March (before the shoot of the stem) to June (flowering stage). As deduced from signal intensities of UV chromatograms, 1,4-, 1,3- and 1,5-diCQA decreased by approximately 30%, the 4,5-isomer even by 80% (data not shown).

In a retention time range of 59 to 70 min, several esters of caffeic, ferulic, sinapic and *p*-coumaric acids were characterized based on their fragmentation patterns. These hydroxycinnamates are known to serve as defence against herbivores and microorganisms, for protection from UV-B radiation as well as response to mechanical damage [26]. Flavonoids such as apigenin and its glucoside have been described as bioactive polyphenols in chamomile flower extracts and decoctions [8]. They were, however, not detected in chamomile roots.

**Figure 5:**
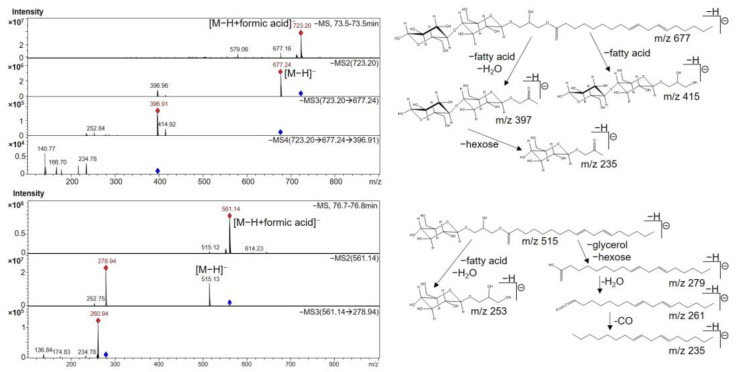
MS^n^ spectra of two glyceroglycolipids (compounds **38** and **41**) and postulated fragmentation pathways.

In the last part of the chromatogram of EtOAc extracts, a number of glyceroglycolipids and phospholipids containing linoleic and linolenic acid moieties were eluted. Interestingly, when linoleic acid diglycosyl monoglyceride (**38**) was fragmented, the fatty acid moiety was released as a neutral loss and the polar head was further fragmented (Figure 5). In contrast, for linolenic and linoleic acid monoglycosyl monoglycerides (**39**–**41**, **43**), the fatty acid served as base peak in MS^2^ experiments and was further fragmented, although the polar head was also detected in the MS^2^ spectrum. Similar representatives of these compound classes have been described in other *Asteraceae* species. For example, glyceroglycolipids have been extracted from dandelion (*Taraxacum mongolicum* L.) [53] and glycerophospholipids from red lettuce (*Lactuca sativa* L. var. *crispa*) leaves and sunchoke (*Helianthus tuberosus* L.) roots [36].

### 2.2. Phytochemical Comparison of Different Chamomile Varieties

Based on the chemical composition of the essential flower oil, chamomile varieties are classified into different chemotypes [9]. In this study, two different cultivars of *M. recutita* and one of *M. discoidea* were compared. In order to determine the chemotypes of the investigated samples, essential flower oil was obtained by aqueous steam distillation and analyzed by GC-MS. The dark blue essential flower oil of *M. recutita* grown in Bad Boll contained equal amounts of the bisabolol oxides A and B. The plant was therefore identified as chemotype D according to Schilcher et al. [9]. In contrast, *α*-bisabolol was the main compound in the essential flower oil of the chamomile cultivar from Sulzemoos, indicating chemotype C [9]. Interestingly, the essential flower oil of pineapple weed (*M. discoidea*) lacked the blue colour and thus chamazulene, but also bisabolol and its oxides. Instead, the terpenes *β*-pinene, *β*-cubebene and *tr*-nerolidol were detected. However, the relationship with other *Matricaria* species was evident from the presence of its main metabolites *β*-farnesene and *cis*-spiroether.

**Figure 6:**
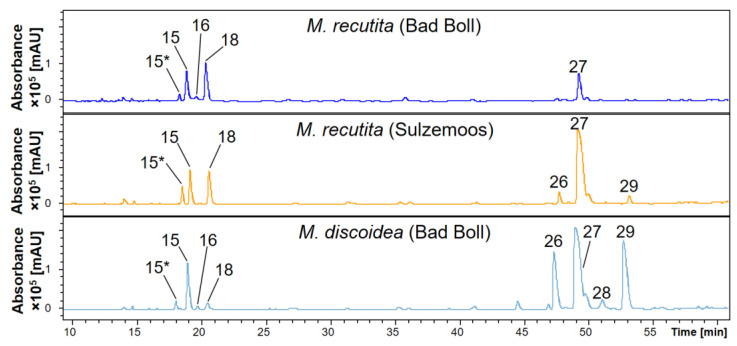
HPLC-DAD UV chromatograms (200–600 nm) showing coumarins and caffeoylquinic acids in *n*-butanol extracts of different chamomile varieties. Peak numbers refer to Table 2. 15: 5-*O*-caffeoylquinic acid (* formic acid adduct); 16: fraxin; 18: isofraxidin-7-hexoside; 26–29: 1,4-/1,3-/1,5-/4,5-dicaffeoylquinic acids.

Contrary to the essential flower oils, the volatile secondary metabolites were identical in DCM root extracts of these three varieties. Chamomillaesters I and II (Figure 2) were identified in all samples along with *β*-farnesene, the unidentified sesquiterpene oxide (Table 1), *cis*- and *trans*-spiroether and the free fatty acids palmitic, linoleic and linolenic acid. Also, the HPLC-DAD-MS^n^ screening of root extracts of increasing polarity revealed similar fingerprints of the three investigated varieties, except for coumarins and caffeoylquinic acids, where differences were particularly apparent. Figure 6 displays the corresponding section of the HPLC UV trace of roots harvested at flowering stage (May/June). Although at this harvest time mainly 1,3-dicaffeoylquinic acid was present in the BuOH extracts of both *M. recutita* cultivars, *M. discoidea* extracts additionally contained the 1,4- and 4,5-isomers in almost equal amounts as deduced from the signal intensities recorded at 320 nm. In contrast, the isofraxidin-7-hexoside content was lowest in *M. discoidea*.

### 2.3. Antioxidant Potential of Chamomile Root Extracts

#### 2.3.1. DPPH Assay

**Figure 7:**
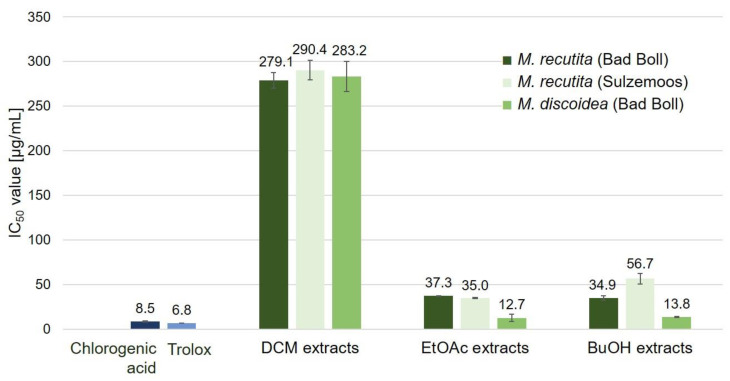
IC_50_ values of different chamomile root extracts, trolox and chlorogenic acid determined applying the DPPH radical scavenging assay (*n* = 3).

The DPPH radical scavenging assay is very common for the determination of antioxidant activities of plant extracts in vitro, although results published in literature may vary due to a lack of standardization and access to different extraction techniques, solvents and chemicals [61]. This assay has been performed in a methanolic solution or on TLC plates assessing various chamomile extracts [36,62,63]. IC_50_ values amounted to 6.8 ± 0.01 µg/mL and 8.5 ± 0.7 µg/mL for the two reference substances trolox and chlorogenic acid, respectively (Figure 7). DCM extracts of the studied chamomile varieties revealed the highest IC_50_ values of 279–290 µg/mL and, thus, the weakest antioxidant activity. *M. discoidea* EtOAc and BuOH extracts had the strongest DPPH scavenging activity with IC_50_ values of 12.7 ± 3.8 and 13.8 ± 0.4 µg/mL, respectively.

Different solvent extracts from aerial plant parts of chamomile have been evaluated with regard to their DPPH scavenging potential in a large number of studies. The IC_50_ values of essential oil and methanol extract of *M. recutita* leaves was reported to be 4.18 μg/mL and 1.83 μg/mL, respectively [64]. Al-Dabbagh and co-workers determined an IC_50_ value of 26.7 µg/mL for a hydroethanolic chamomile flower extract [65]. Subcritical water extracts of chamomile flowers revealed IC_50_ values of 10–45 µg/mL, depending on the extraction temperature [66]. Thus, the IC_50_ values determined in our study for EtOAc and BuOH extracts are in the same range as those of the flowers. Generally, infusions and decoctions, i.e., aqueous solutions, possess higher antioxidant activities than methanol extracts [45] and IC_50_ values decrease with the increasing polarity of the solvent used [62]. Accordingly, in this study, extracts of increasing polarity showed lower IC_50_ values, indicating stronger antioxidant properties.

Phenolic acids and flavonoids have been identified as main contributors to the antioxidant activity of various chamomile extracts [6]. The radical scavenging effect of ethyl acetate and butanol extracts is probably due to coumarins and the abundant mono- and diCQA derivatives identified by HPLC-DAD-MS^n^. The stronger effect of *M. discoidea* extracts may be due to the fact that they contain higher amounts of 1,4- and 4,5-dicaffeoylquinic acids than the *M. recutita* cultivars (Figure 6), and the 4,5-isomer has been shown to have strongest DPPH scavenging activity among the diCQA isomers [67]. However, a direct comparison of reference substances with plant extracts, which are complex mixtures of numerous metabolites, remains challenging, since synergistic, additive or antagonistic effects may also affect the final read-out values.

#### 2.3.2. Superoxide Assay

**Figure 8:**
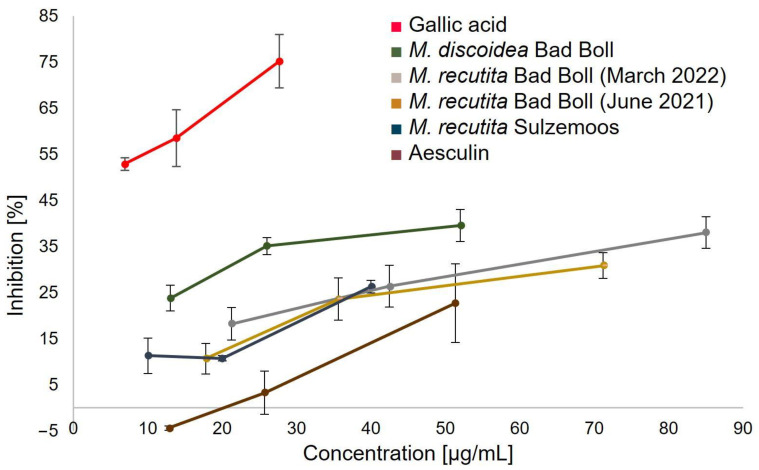
Superoxide anion radical scavenging activity of various chamomile root butanol extracts, aesculin and gallic acid. Results represent mean ± SD (*n* = 3). Negative inhibition values result from mathematical calculation of the relative inhibition.

The superoxide radical O_2_^●−^ belongs to the reactive oxygen species (ROS) and is generated in cells by mitochondrial electron transfer systems, NADPH oxidase and xanthine oxidase. Consequently, antioxidants and radical scavenging enzymes, which protect cells from oxidative stress, are crucial for preventing adverse effects such as increased ageing and Alzheimer’s disease [68]. In contrast to the DPPH assay, the superoxide assay is performed under physiological conditions. This allows a better understanding of the antioxidant effects of chamomile root extracts in vivo. Although strong antioxidant activities have been determined for caffeoylquinic acids in general [69], chlorogenic acid as pure compound did not show any effect in this assay. Since trolox also had no effect, gallic acid was used as reference substance. Additionally, aesculin, one of the coumarins detected in the extracts, was tested as second reference substance. Due to the insufficient solubility of DCM and EtOAc extracts in the buffer solution, which led to turbidity, only BuOH extracts were investigated. The relative inhibition of formazan formation by the different samples is displayed in Figure 8. The least amount of formazan was formed in samples containing 5–30 µg/mL gallic acid, thus absorbances remained low and relative inhibition was highest. This indicates that among all samples gallic acid had the strongest superoxide scavenging activity. This is in accordance with the findings of Furuno et al., that the pyrogallol moiety strongly contributes to superoxide radical scavenging activity [70]. In comparison, BuOH extracts showed moderate superoxide scavenging activity. Similar to the DPPH assay, *M. discoidea* exerted the most pronounced antioxidant effects among the chamomile samples studied. The different *M. recutita* samples showed similar results, regardless of origin or harvest date. Aesculin as reference standard showed lowest inhibition and, thus, a very weak superoxide scavenging effect. The extracts studied are complex mixtures whose antioxidant effects are probably caused by the sum of their individual components such as gallic acid and other phenolic acids, coumarins and further metabolites.

The superoxide scavenging activity of chamomile has not been widely assessed. Merely Cvetanovic et al. determined IC_50_ values between 30 and 100 µg/mL in electron spin resonance (ESR) studies [71]. Physiological antioxidant effects of chamomile flower essential oil and extracts have been investigated in different studies. As an example, Sebai et al. tested the impact of chamomile flower decoction against oxidative stress in rats. The authors showed, that chamomile decoction protected the animals from castor oil-induced diarrhea and intestinal fluid accumulation but also prevented the reduction of the activity of antioxidant enzymes such as catalase and superoxide dismutase [72]. Accordingly, administration of chamomile flower decoction protected these enzymes from ethanol-induced injury and prevented lipoperoxidation in the liver [73]. The effects were attributed to phenolic compounds, which also occur in chamomile roots.

Antioxidant properties are desired not only in medicinal applications, but also in the cosmetics and food sector. Many slightly or more highly processed products require the addition of stabilizing, coloring or preserving ingredients [74]. However, there is a growing consciousness for natural formulations without synthetic additives. Therefore, plant extracts, e.g., rosemary essential oil, are increasingly incorporated as natural antioxidant compounds in different food and cosmetic matrices [75,76]. In the case of German chamomile, research is again focused on extracts or essential oils from flowers or above-ground plant parts, e.g., to enhance the stability of dairy products without changing their nutritional value [77]. In the light of the present study, root extracts with their comparably potent antioxidant activity should also be considered in the future.

### 2.4. Antibacterial Potential of Chamomile Roots

Due to increasing resistance to conventional antibiotics, the use of natural products for their supplement or substitution is a promising research topic [26,62]. For a first evaluation of the potential antibacterial activity of different *M. recutita* root extracts, disk diffusion experiments were performed. All samples inhibited the growth of Gram-positive bacterial strains of *B. subtilis* and *S. aureus* in amounts ≥ 0.8 mg per disk, as shown in Table 3. Antibacterial effects were comparable for both susceptible strains. Except for *M. recutita* grown in Bad Boll, DCM and EtOAc extracts showed stronger inhibition than BuOH extracts. This is not surprising since the antibacterial effects of many essential oils have already been described [78] and the main constituents of these, e.g., terpenoids, are also present in nonpolar extracts. Interestingly, DCM and EtOAc root extracts of *M. discoidea* showed the strongest effects, but the corresponding BuOH extract was completely inactive. Inhibiting effects could neither be detected against Gram-negative bacteria strains *P. aeruginosa* and *E. coli* nor against *C. albicans* (data not shown).

The antibacterial effects of various compound classes are based on different mechanisms. Essential oil constituents such as terpenes can pass or interact with bacterial cell membranes, which may go along with disruption or leakage. Inside the cells, oxidative stress and disturbance of protein metabolism and mitochondria may occur, among others [79]. Cinnamic and chlorogenic acids are also known to disrupt bacterial cell membranes, thus increasing their fluidity and permeability [80]. Furthermore, some coumarins have been reported to inhibit DNA gyrase, which normally causes negative supercoiling of the DNA [81].

Although the antimicrobial potential of chamomile flowers has been extensively studied, information about the roots is scarce. An antibacterial potential of chamomile roots has been described against *Pseudomonas syringae* pv. *maculicola*. The effects could be attributed to the presence of spiroethers and coumarins, but have not been studied further [82]. In contrast, roots of other members of the *Asteraceae* family have been assessed in more detail. For example, dandelion (*Taraxacum officinale* L.) roots inhibited *S. aureus* and *B. cereus* growth, presumably due to the presence of hydroxylinoleic and hydroxylinolenic acids, vanillin and coniferylaldehyde [83]. The inhibitory effect of tansy (*Tanacetum vulgare* L.) root extracts against *B. subtilis* and two plant pathogens could be attributed to different polyacetylenic compounds [63].

A lipophilic chamomile flower extract obtained by supercritical CO_2_ extraction inhibited the growth of different crop-borne fungi by 80–100% [84]. Roby et al. compared the antibacterial potential of different chamomile flower extracts. Consistent with all other studies, the extracts were more effective against Gram-positive than against Gram-negative bacteria. Very low amounts of 7.5–20 µg per disk inhibited the growth of various bacterial strains and *C. albicans* [62]. Higher concentrations were used by Abdoul-Latif et al.: 300 µg leaf methanol extract or 10 µL essential oil per disk inhibited the growth of different Gram-positive and Gram-negative bacterial strains, with the essential oil showing stronger effects [64]. Interestingly, bisabolol oxides negatively influenced antibacterial activity [4], indicating that the activity strongly depended on the compound profile of the respective sample. Thus, for an appropriate use, the chemotype of the essential flower oil as well as season of harvest and the extraction procedure have to be chosen carefully [85]. The presented results show that, in addition to chamomile flowers and leaves, the roots also have promising potential with regard to their antibacterial properties. Thus, the use of chamomile roots for the preparation of phytomedicinal products contributes to a sustainable cultivation and use of this important medicinal plant, although further studies are needed, e.g., to determine minimal inhibitory concentrations of the respective extracts, allowing a profound assessment of the antibacterial potential.

## 3. Materials and Methods

### 3.1. Chemicals and Reagents

Acetone, acetonitrile, *n*-butanol (BuOH), dichloromethane (DCM), dimethylsulfoxide (DMSO), chloroform, ethanol, ethyl acetate (EtOAc), methanol (MeOH) and toluene were purchased from Chemsolute (Th. Geyer GmbH & Co., KG, Renningen, Germany). Nitrotetrazolium blue chloride (NBT), gallic acid monohydrate and TRIS hydrochloride were obtained from Carl Roth GmbH & Co., KG (Karlsruhe, Germany). *N*,*O*-Bis (trimethylsilyl)-trifluoroacetamide (BSTFA), 2,2-diphenyl-1-picrylhydrazyl (DPPH), hypoxanthine, tryptophane and xanthine oxidase (XOD, grade III from bovine milk) were from Sigma-Aldrich (St. Louis, MO, USA), and formic acid from Fluka (Sigma Aldrich, St. Louis, MO, USA). Trolox was purchased from Cayman Chemical Company (Ann Arbor, MI, USA), and chlorogenic acid hemihydrate from Alfa Aesar (Karlsruhe, Germany). Fraxin and aesculin analytical standards were obtained from PhytoLab GmbH & Co., KG (Vestenbergsgreuth, Germany). *N*,*N*-Dimethylformamide (DMF), sodium sulfate, Tryptic Soy Agar (TSA) and Sabouraud Dextrose Agar (SDA) broth and agar plates were from Merck KGaA (Darmstadt, Germany). Ampicillin sodium salt and gentamicin were from Sigma-Aldrich Chemie GmbH (Steinheim, Germany).

### 3.2. Plant Material and Extraction

Roots of *M. recutita* were harvested monthly between March and June 2021 and in March 2022 in the medicinal plant garden of WALA Heilmittel GmbH (Bad Boll/Eckwälden, Germany). Further, roots of *M. discoidea* were harvested in the same place in June 2021. Additionally, roots of a bisabolol-rich *M. recutita* variety were harvested at Kistler & Co., GmbH in Sulzemoos, Germany, in June 2021. The plant material was rinsed with tap water, drained, packed in freezer bags and stored at −80 °C until investigation. Voucher specimens were deposited at the herbarium of the Institute of Botany, Hohenheim University (Stuttgart, Germany). The identity of the plant material was confirmed by Dr. R. Duque-Thüs (*M. recutita* Bad Boll, voucher number: HOH-022871; *M. recutita* Sulzemoos, voucher number: HOH-022870; *M. discoidea* Bad Boll, voucher number: HOH-022872).

100 g of fresh plant material was mixed with acetone/water (500 mL, 60/40, *v*/*v*). The material was minced for three min using an Ultra-Turrax (17,000 rpm; IKA Werke GmbH and Co., KG, Staufen, Germany). Prior to and after comminution, the mixture was bubbled with nitrogen for 15 min to avoid oxidative degradation of the plant constituents. The slurry was stored at 4 °C overnight and then filtered over Celite^®^ (Carl Roth GmbH + Co., KG, Karlsruhe, Germany). Solid residues were extracted a second time in the same manner. Both brown-coloured filtrates were combined and acetone was removed by rotary evaporation.

Subsequently, the obtained aqueous extract was successively extracted with 3 × 100 mL each of dichloromethane, ethyl acetate and *n*-butanol, using a separating funnel. Dichloromethane and ethyl acetate extracts were dried over anhydrous sodium sulfate and filtered over a glass frit (Por. 3, ROBU^®^ Glasfilter-Geräte GmbH, Hattert, Germany). The solvents were then removed in vacuo to obtain dry extracts for further investigations. Extraction was performed in duplicate for all three chamomile species.

Additionally, 50 g of either chamomile roots or flowers, stems and leaves in 200 mL water were distilled in a Clevenger-type apparatus for four hours. Essential oils were trapped in *n*-hexane/ethyl acetate 3/1 (*v*/*v*) and dried over anhydrous sodium sulfate.

### 3.3. GC-MS Analysis of Volatile Constituents

Crude extracts obtained by solvent extraction were dissolved in chloroform at concentrations of 5 mg/mL for direct analysis. Essential oils in *n*-hexane/ethyl acetate, recovered upon distillation as described above, were directly injected into the GC. To obtain trimethylsilyl derivatives of individual compounds, crude extracts (3–5 mg) were dissolved in DMF (500 µL) and 200 µL BSTFA were added. The solution was heated to 105 °C for 15 min and subsequently analyzed via GC/MS.

GC/MS analyses were conducted with a PerkinElmer *Clarus 500* gas chromatograph (PerkinElmer, Inc., Shelton, CT, USA) with split injection (split ratio 30:1, injection volume 1.0 μL) coupled to a single quadrupole mass spectrometer operating in electron ionization (EI) mode at 70 eV. A *Zebron ZB-5MS* capillary column (60 m × 0.25 mm i.d., 0.25 μm film thickness, 5% phenylpolysiloxane and 95% dimethylpolysiloxane coating; Phenomenex, Torrance, USA) was used as a stationary phase, helium served as carrier gas at a flow rate of 1 mL/min. The injector temperature was 250 °C, the temperature program of the column oven was 100–320 °C, applying a linear gradient of 4 °C/min and a final holding time of 30 min. Data were acquired and processed using the software *TurboMass* (v.5.4.2, PerkinElmer, Inc., Waltham, MA, USA).

### 3.4. RP-HPLC-DAD-ESI-MS^n^ Analysis

High performance liquid chromatographic analyses were carried out on an Agilent 1200 HPLC system (Agilent Technologies, Inc., Palo Alto, CA, USA) equipped with binary pump, micro vacuum degasser, autosampler, thermostatic column compartment and UV/VIS diode array detector (DAD). A *Kinetex*^®^ C18 reversed-phase column (2.6 μm particle size, 150 mm × 2.1 mm i.d., Phenomenex Ltd., Aschaffenburg, Germany) and a pre-column of the same material were used for chromatographic separation at 25 °C and a flow rate of 0.21 mL/min. The mobile phase consisted of 0.1% formic acid in water (eluent A) and acetonitrile (eluent B). The injection volume of each sample was 10 μL. The gradient was as follows: 0–8 min, 0–10% B; 8–20 min, 10% B; 20–51 min, 10–23% B; 51–70 min, 23–60% B; 70–80 min, 60–100% B; 80–85 min, 100% B; 85–90 min, 100–0% B; 90–100 min, 0% B.

The LC system was coupled to an *HCTultra* ion trap mass spectrometer (Bruker Daltonik GmbH, Bremen, Germany) with an ESI source. All extracts were analyzed in negative ionization mode using a capillary voltage of 4000 V, a dry gas (N_2_) flow of 9.00 L/min with a capillary temperature of 365 °C and nebulizer pressure of 35 psi. Full scan mass spectra (mass range *m*/*z* 50–1000) of HPLC eluates were recorded during chromatographic separation yielding [M–H]^−^ ions. MS^n^ data were acquired in the auto MS/MS mode by collision-induced dissociation (CID). The instruments were controlled by *ChemStation for LC 3D systems* (Rev. B01.03 SR1 (204)) and *EsquireControl* software (V7.1).

Samples were dissolved in water (BuOH extracts) or methanol (all other extracts) to reach a concentration of 5 mg/mL.

### 3.5. 2,2- Diphenyl-1-picrylhydrazyl (DPPH) Assay

The DPPH free radical scavenging assay is based on the ability of antioxidant components to reduce the artificial stable DPPH radical, going along with a change of colour from deep purple to yellow and, thus, a strong decrease in absorbance at 516 nm. The half maximal inhibitory concentration (IC_50_) is the amount of sample needed to reduce the initial DPPH content by 50% and an indication of the antioxidant potential of individual compounds or complex plant extracts. For the assay, DPPH was dissolved in methanol at a concentration of 100 mM. The plant extracts were dissolved at concentrations of 1–4 mg/mL in methanol and diluted to five appropriate concentrations. Then, 200 µL of the test or reference solution or methanol as blank sample were added to 1800 µL DPPH solution. The sample was incubated at 38 °C for 30 min and then analyzed at 516 nm using a spectrophotometer (Lambda 2, Perkin Elmer Ltd., Waltham, MA, USA) as reported previously [86]. Trolox was used as reference compound preparing solutions at five different concentrations ranging from 3–100 mM. Absorbance values for each sample were plotted against the concentrations, and IC_50_ values were calculated from the formula of the linear trend line at 50% of the maximum absorbance value. Analyses were performed in triplicate.

### 3.6. Superoxide Assay

The ability of BuOH extracts to scavenge the superoxide radical O_2_^●-^ was investigated using a modified version of the procedure described by Lorenz et al. [87]. Superoxide was generated enzymatically using a hypoxanthine/xanthine oxidase (XOD) system and analyzed by the reduction of NBT to form a blue formazan product. The latter was detected using a spectrophotometer (Lambda 2, Perkin Elmer Ltd., Waltham, MA, USA). 50 mM TRIS buffer at pH 7.4 containing 539 µM hypoxanthine and 111 µM NBT was used as solvent. Solid BuOH root extracts were dissolved in DMSO and diluted to three different concentrations in the range of 1–9 mg/mL. Subsequently, 1960 µL buffer solution was mixed with 20 µL sample solution and 20 µL enzyme solution (3.4 U/mL). Samples were incubated at 37 °C for exactly 7 min after enzyme addition and immediately analyzed spectrophotometrically at 560 nm against a blank control not containing the enzyme. Gallic acid and aesculin were used as reference compounds. Analyses were performed in triplicate. The percentage inhibition of formazan formation was calculated using the following equation:

Inhibition (%) = (A_control_ − A_sample_)/A_control_ × 100

where A_control_ and A_sample_ were the absorbance values of the control solution with pure DMSO and the sample solution, respectively.

### 3.7. Antimicrobial Assay

Disk diffusion tests were performed to evaluate the antimicrobial activity of different chamomile root extracts against four common bacteria strains. Among these, Gram-negative strains, i.e., *Pseudomonas aeruginosa* ATCC 9027 and *Escherichia coli* ATCC 8739, and Gram-positive strains, i.e., *Staphylococcus aureus* ATCC 6538 and *Bacillus safensis* ATCC 6633 were tested (Leibniz Institute, DSMZ-German Collection of Microorganisms and Cell Cultures GmbH, Braunschweig, Germany). Additionally, one fungal strain (*Candida albicans* strain ATCC 10231) was tested. Cell material was taken from pure cultures and incubated in 4 mL TSA broth (bacteria) or SDA broth (*C. albicans*) at 37 °C for 24 h. Colony-forming units were determined by serial dilution to 10^6^–10^8^ (*B. safensis*, *E. coli*, *C. albicans*) and 10^9^ (*S. aureus, P. aeruginosa*). Thus, the latter were diluted with TSA broth (1:10, v:v) prior to usage. Plant extracts were suspended in MeOH at a concentration of 80 mg/mL. Sterile antimicrobial test disks (Oxoid™ blank, Thermo Fisher Diagnostics GmbH, Waltham, MA, USA) were loaded with 10–40 µL suspension (0.8–3.2 mg dry extract) and dried. Pure MeOH (10 µL) served as negative control, and the antibiotics gentamicin (0.5 mM, 1.0 mM, 1.5 mM; 10 µL) and ampicillin (0.1 mM, 10 µL) were used as positive controls for *S. aureus, E. coli, P. aeruginosa* and *B. safensis*, respectively. Subsequently, 100 µL of the bacterial suspension was spread on a TSA agar plate and allowed to dry briefly. SDA agar plates were used for *C. albicans*. Disks with negative and positive controls as well as three extract concentrations were placed on each plate. Inhibition zones (diameter in millimeter including the test disk) were measured after incubation at 37 °C for 20 h. The assay was conducted in triplicate for all samples.

## 4. Conclusions

In the present study, the roots of two *Matricaria recutita* and one *M. discoidea* accessions were investigated for their secondary metabolite composition and bioactivity characteristics. Interestingly, although the volatile constituents in essential flower oils varied considerably between the three varieties, all roots contained similar principal constituents. Among others, *β*-farnesene, chamomillol, spiroether and chamomillaester were detected by GC-MS. Additionally, HPLC-DAD-MS^n^ analyses revealed the presence of the coumarin glycosides aesculin, scopolin, fraxin and isofraxidin-7-hexoside along with other coumarin derivatives, caffeoylquinic acids, phospho- and glyceroglycolipids in the roots.

EtOAc and BuOH root extracts showed a DPPH radical scavenging activity comparable to that of chamomile flowers. Thus, middle polar extracts may be incorporated into emulsions or oil-based cosmetic products to improve their stability and antioxidant properties. The BuOH extracts also had scavenging effects on the superoxide (O_2_^●−^) radical when evaluated under physiological conditions in buffered solution at pH 7.4. This may point to an antioxidant potential of the extracts in vivo. Moreover, moderate antibacterial activity of chamomile root extracts against the Gram-positive bacterial strains *S. aureus* and *B. subtilis* was observed. Chamomile roots are a by-product of chamomile tea and essential oil production. Their use in phytomedicinal or cosmetic preparations thus contributes to a more sustainable agricultural production. However, the efficacy of such preparations should be evaluated in further studies.

## Figures and Tables

**Table 1:** Volatile compounds in *M. recutita* root DCM extracts assigned based on their GC-MS characteristics. Base peaks are displayed in bold.

No.	Compound	t_R_ (min)	MW (g/mol)	*m*/*z* (M^+^ Int. %)
1	Berkheyaradulene	17.9	204	204 (15%), 189, **162**, 147, 134, 119
2	*β*-Farnesene	19.2	204	204 (10%), 161, 133, 120, 107, 93, 79, **69**, 55
3	*α*-Farnesene	20.8	204	204 (1%), 161, 119, 107, **93**, 79, 69, 55
4	Neryl-isovalerate	23.6	238	238 (1%), 136, 121, 107, 93, 85, **69**, 57
5	Chamomillol	25.1	222	222 (10%), 204, 179, 161, **119**, 105, 81
6	*Not identified*	30.7	220	**220** (100%), 190, 178, 136
7	*cis*-Spiroether	31.6	200	**200** (100%), 170, 157, 128, 115,76
8	*trans*-Spiroether	31.8	200	**200** (100%), 170, 157, 128, 115,76
9	Palmitic acid *	34.9	328	328 (20%), 313, 145, 161, 117, 73, 55
10	Chamomillaester I	35.6	228	228 (20%), 168, **153**, 141, 128, 115, 91, 77
11	Chamomillaester II	37.2	228	228 (25%), 168, **152**, 141, 128, 115, 91, 77
12	Linoleic acid *	38.7	352	352 (10%), 337, 262, 220, 129, 81, **73**, 67
13	Linolenic acid *	38.8	350	350 (10%), 335, 157, 129, 108, 95, **73**, 55

* Trimethylsilyl ester.

**Table 2:** HPLC-DAD-MS^n^ data of compounds detected in ethyl acetate and *n*-butanol extracts of *M. recutita* roots in negative ionization mode.

EtOAc Extract (A) ^a^	BuOH Extract (C) ^a^	t_R_ (min)	Substance	UV Maxima (nm) ^b^	Mass Spectrometric Data (*m*/*z*) ^c^			Reference
					MS^1^	MS^2^	MS^3^	
1		1.7	Chlorogenic acid hexoside	234, 324	**515**	353	191, 135	[29]
	2	1.9	Sucrose	-	683, **533**, 439, **404**	**341**, 179	143	[30]
	3	2.4	1-Kestose	-	**637**, 549, 503	**503**, **464**, 323		[30]
	4	3.7	Uridine	202, 262	**243**	**200**, 152	138, 110	[31]
	5	7.3	*trans*-Zeatin riboside	204, 258	533, **312**	**266**, 134	134	MassBank PR100614
	6	9.9	Ellagic acid	ND ^d^	**347**	**301**	223, 161, 139	[32]
	7	12.8	Galloyl hexoside	ND ^d^	**331**	**169**, 161	152, 139	[33]
	8	13.5	Galloyl-3-*O*-*β*-D-glucuronide	ND ^d^	**391**	**345**, 207, 183	331, 183	[34]
	9	14.0	*L*-Tryptophan	220, 278	**203**	**159**, 158		[35,36], standard
	10	14.6	3-*O*-Caffeoylquinic acid	324	**353**	**191**, 179, 135	85	[37,38]
	11	15.1	Fraxin sulfate	206, 230, 288	**449**	**369**, 241	207, 192	[39]
	12	15.6	Aesculin	290 sh, 342	**339**	**177**	133	[40,41], standard
	13	16.4	Caffeoyl-Fraxetin	259, 305	**387**, 339	207, **179**	164, 161, 146	Tentative
	14	18.4	Scopolin	205, 226, 288 sh, 338	443, 419, **399**	353, 237, **191**, 176	176	[42]
15	15	18.9	5-*O*-Caffeoylquinic acid	218, 235 sh, 290 sh, 324	707 *	**353**	191, 173, 135	[43], standard
16	16	19.7	Fraxin	208, 230, 300	**369**, 221	**207**	192	[42], standard
	17	19.9	Fraxetin sulfate	206, 230, 338	**287**	**207**	192	[39]
18	18	20.6	Isofraxidin-7-hexoside	208, 228 sh, 294, 334 sh	**429**, 383, 287, 221	**221**	206, 191	[42]
	19	22.6	4-*O*-Caffeoylquinic acid	324	**353**	191	173, 93	[44]
	20	26.7	Fraxetin derivative	ND ^d^	**585**	**377**	**329**, 314	Tentative
21		35.4	3,5-Dicaffeoylquinic acid (3,5-diCQA)	218, 236, 322	533, **515**	**353**, 335	191, 179, 135	[44]
	22	35.9	Ferulic acid hexoside	223 sh, 236, 295 sh, 318	**711**	**355**	193, 149	[45,46]
	23	37.7	Acetylquinic acid	ND ^d^	489, **233**	171, 143, 127		[40]
	24	41.1	Dimethyl lithospermate	226, 276	**565**, 467	**339**, 327	323, 309, 294	[47]
	25	43.9	Tricaffeoyl-quinic acid	322	**677**	515, **353**	191, 179, 135	[48]
	26	47.4	1,4-diCQA	218, 242, 300sh, 324	**515**	**353**, 335	191, 179, 173, 135	[44,46]
	27	49.1	1,3-diCQA	218, 236 sh, 300 sh, 326	**515**	**353**, 191	191, 179, 135	[44]
28	28	49.5	1,5-diCQA	218, 242, 300 sh, 326	**515**	**353**, 335, 191	191, 179, 135	[44]
29	29	52.8	4,5-diCQA	220, 242, 300 sh, 326	**515**	**353**, 203	191, 179, 173, 135	[44]
30	30	58.5	3,4-diCQA	280, 322	**515**, 439, 345	**353**, 191, 173	191, 179, 173, 135	[6,44]
31		59.2	Caffeoyl-feruloylquinic acid	328	**529**, 439	**367**, 349	334, 191, 179, 161	[40,49]
	32	59.3	unknown	ND ^d^	**439**	393, **379**	349, 235, 217	
	33	59.9	Chicoric acid (acetyl derivative)	ND ^d^	**515**, 455	473, 353, 311, **263**, 221, 179	203, 179, 161, 143	[36,50]
	34	60.7	Caffeic acid derivative	ND ^d^	707, **519**	**477**	263, 221, 179, 161	Tentative
35		61.8	Sinapoyl-feruloyl-caffeoylquinic acid	242, 328	**735**	**559**	517, 337, 235, 193	[32,51]
36		66.9	Coumaroyl-feruloylquinic acid	238, 324	**707**, 427	**513**, 367	**367**, 173	[48]
37		69.9	Diferuloylquinic acid	242, 318	707, **645**	**543**	367	[48,49]
38		73.5	Linoleic acid diglycosyl monoglyceride	228, 238, 316	**723**	**677**, 397	415, 397, 235	[35]
39, 40		74.8 75.4	Linolenic acid monoglycosyl monoglyceride isomers	240, 313	**559**	513, **277**, 253	259, 233	[35,52]
41		76.8	Linoleic acid monoglycosyl monoglyceride	238, 250, 314	**561**	515, **279**	261, 205	[35,53]
42		77.0	Linoleic acid derivative	238, 314	**529**	511, **279**, 249	261, 205	Tentative
43		77.3	Linoleic acid monoglycosyl monoglyceride	240, 316	**561**	515, **279**	261, 205	[35,53]
44		77.8	Linoleic acid derivative	242, 254, 324	**529**	511, **279**, 249	261, 205	Tentative
45, 46		78.0 78.4	Phosphoglyceride isomers	242, 250, 324	**431**	171, **153**	97, 79	[52]
47		82.1	Phosphoglyceride	314	**433**, 399	171, **153**	79	[52]
48		82.5	Linolenic acid	<200, 242	311, **277**	259, **233**, 205	191, 179	[35,54]
49		84.2	Linoleic acid	<200	**279**	**261**	243	[35,54]
50		85.7	Dihydroxy-linolenic acid	226	**325**, 281	**183**		[35], tentative

^a^ For peak labeling see Figure 3; ^b^ UV and BPC intensities may differ due to differences in analyte ionizability, concentrations, molar extinction coefficients, etc.; ^c^ bold numbers: ion further fragmented in CID experiments; ^d^ not detected; * dimer is an artifact produced during ionization.

**Table 3:** Mean inhibition zones in mm against Gram-positive bacterial strains of *B. subtilis* and *S. aureus* (*n* = 3).

	*S. aureus*			*B. subtilis*		
Extract	0.8 mg/Disk	1.6 mg/Disk	3.2 mg/Disk	0.8 mg/Disk	1.6 mg/Disk	3.2 mg/Disk
*M. recutita* Bad Boll						
DCM	8 ± 0	9 ± 0	9 ± 2	6 ± 0	7 ± 0	8 ± 0
EtOAc	8 ± 0	7 ± 1	9 ± 1	-	8 ± 1	10 ± 1
BuOH	7 ± 0	8 ± 1	10 ± 1	7 ± 1	8 ± 0	9 ± 1
*M. recutita* Sulzemoos						
DCM	7 ± 1	9 ± 1	10 ± 1	8 ± 0	9 ± 0	9 ± 1
BuOH	-	8 ± 1	9 ± 1	-	7 ± 0	8 ± 1
*M. discoidea*						
DCM	9 ± 1	10 ± 1	11 ± 1	9 ± 2	9 ± 2	9 ± 1
EtOAc	7 ± 0	9 ± 1	10 ± 0	8 ± 0	9 ± 0	10 ± 0
BuOH	-	-	-	-	-	-

## Data Availability

Not applicable.

## Sample Availability

Not available.
